# Supplementary material for: Learning steers the ontogeny of an efficient hunting sequence in zebrafish larvae
Source: eLife. 2020 Aug 10;9:e55119. doi: 10.7554/eLife.55119 (PMC7561354; doi:10.7554/eLife.55119)
Supplement: Transparent reporting form [file elife-55119-transrepform.docx]

***eLife’s* transparent reporting form**

We encourage authors to provide detailed information *within their submission* to facilitate the interpretation and replication of experiments. Authors can upload supporting documentation to indicate the use of appropriate reporting guidelines for health-related research (see [EQUATOR Network](http://www.equator-network.org/ \t _blank)), life science research (see the [BioSharing Information Resource](https://biosharing.org/)), or the [ARRIVE guidelines](http://www.plosbiology.org/article/info:doi/10.1371/journal.pbio.1000412) for reporting work involving animal research. Where applicable, authors should refer to any relevant reporting standards documents in this form.

If you have any questions, please consult our Journal Policies and/or contact us: [editorial@elifesciences.org](mailto:editorial@elifesciences.org).

**Sample-size estimation**

- You should state whether an appropriate sample size was computed when the study was being designed
- You should state the statistical method of sample size computation and any required assumptions
- If no explicit power analysis was used, you should describe how you decided what sample (replicate) size (number) to use

Please outline where this information can be found within the submission (e.g., sections or figure legends), or explain why this information doesn’t apply to your submission:

**Replicates**

- You should report how often each experiment was performed

Sample size for group hunting efficiency see Mat.Methods, Behavioural recording, ln. 613.

- You should include a definition of biological versus technical replication
- The data obtained should be provided and sufficient information should be provided to indicate the number of independent biological and/or technical replicates
- If you encountered any outliers, you should describe how these were handled
- Criteria for exclusion/inclusion of data should be clearly stated
- High-throughput sequence data should be uploaded before submission, with a private link for reviewers provided (these are available from both GEO and ArrayExpress)

Please outline where this information can be found within the submission (e.g., sections or figure legends), or explain why this information doesn’t apply to your submission:

**Statistical reporting**

Experimental time-frame and replicates, see Mat.Methods, Behavioural recording, paragraph starting ln. 609.

Inclusion criteria for larvae behaviour recordings, see Mat.Methods, Behavioural recording ln 606

- Statistical analysis methods should be described and justified
- Raw data should be presented in figures whenever informative to do so (typically when N per group is less than 10)
- For each experiment, you should identify the statistical tests used, exact values of N, definitions of center, methods of multiple test correction, and dispersion and precision measures (e.g., mean, median, SD, SEM, confidence intervals; and, for the major substantive results, a measure of effect size (e.g., Pearson's r, Cohen's d)
- Report exact p-values wherever possible alongside the summary statistics and 95% confidence intervals. These should be reported for all key questions and not only when the p-value is less than 0.05.

Please outline where this information can be found within the submission (e.g., sections or figure legends), or explain why this information doesn’t apply to your submission:

(For large datasets, or papers with a very large number of statistical tests, you may upload a single table file with tests, Ns, etc., with reference to sections in the manuscript.)

We explain our statistical methods : in Results section ln. 103 and in Materials and Methods, section Bayesian statistics and notation (ln. 700)

**Group allocation**

- Indicate how samples were allocated into experimental groups (in the case of clinical studies, please specify allocation to treatment method); if randomization was used, please also state if restricted randomization was applied
- Indicate if masking was used during group allocation, data collection and/or data analysis

Please outline where this information can be found within the submission (e.g., sections or figure legends), or explain why this information doesn’t apply to your submission:

**Additional data files (“source data”)**

- We encourage you to upload relevant additional data files, such as numerical data that are represented as a graph in a figure, or as a summary table

Rearing – embryos are randomly allocated to the three rearing groups see Materials Methods, Rearing (ln 547).

Recording ln: Mat. Methods, Behavioural recording ln. 574

Hunt outcome labelling/scoring blind to group: Mat. Methods,Detecting hunt events and labelling, ln.668

- Where provided, these should be in the most useful format, and they can be uploaded as “Source data” files linked to a main figure or table
- Include model definition files including the full list of parameters used
- Include code used for data analysis (e.g., R, MatLab)
- Avoid stating that data files are “available upon request”

Please indicate the figures or tables for which source data files have been provided:

Tables of presented data are publicly available on the github repository in R rds data format:

https://github.com/kostasl/ontogenyofhunting_pub
but also uploaded as plain CSV files with this submission.

The attached data CSV data belongs to figures :

Fig 2. Hunt rates and duration

Fig. 3 Capture efficiency,

Fig. 4 Capture bout clustering

Fig. 5 First turn to prey behaviour

Fig. 6 Capture speed and turn-ratio

Model statistical model R scripts are also provided on the github repository.
